# MicroRNAs-mRNAs Expression Profile and Their Potential Role in Malignant Transformation of Human Bronchial Epithelial Cells Induced by Cadmium

**DOI:** 10.1155/2015/902025

**Published:** 2015-10-04

**Authors:** Qun Liu, Chanjiao Zheng, Huanyu Shen, Zhiheng Zhou, Yixiong Lei

**Affiliations:** School of Public Health, Guangzhou Medical University, Guangzhou 511436, China

## Abstract

*Background*. Our study was designed to elucidate whether there were miRNA and mRNA aberrantly expression profiles and potential role in malignant transformation of 16HBE induced by Cd. *Methods*. mRNA and miRNA expression profiles were determined in 35th Cd-induced 16HBE and untreated 16HBE by microarray. A series of bioinformatics analyses such as predicting targets, GO, KEGG were performed to find DEGs, coexpressing networks between miRNAs and mRNAs and its functions. *Results*. 498 DEGs were found. 8 Cd-responsive novel miRNAs predicted previously were identified, and 5 of them were downregulated. 214 target genes were predicted for the Cd-responsive miRNAs, many of which appeared to regulate gene networks. Target gene CCM2 was showed reciprocal effect by miRNAs. According to the combination analysis, hsa-miR-27b-3p regulated most of the mRNAs, especially upregulated expression genes. The differentially expressed miRNAs are involved in the biological processes and channels, and these GO and KEGG enrichment analyses result were significantly enriched in the Cd-responsive. *Discussion*. These results provided a tight link for the miRNA-mRNA integrated network and implied the role of novel miRNAs in malignant transformation of 16HBE induced by Cadmium. It is better to understand the novel molecular mechanism of cadmium-induced tumorigenesis.

## 1. Introduction

Cadmium (Cd) is a heavy metal that is widely applied in industry. However, its toxicity affects human health through occupational and environmental exposure [1–3]. High levels of Cd can be accumulated in various organs, and toxicological responses to Cd exposure include lung, kidney, and liver damage [4–6]. Several lines of experimental evidence have shown that Cd is carcinogenic to human and animals [7]. Based on epidemiology and laboratory evidences, Cd and its compounds were classified as human carcinogens in 1993 by the International Agency for Research on Cancer (IARC) [8].

The molecular mechanism is well understood in the etiology of heavy metal-associated diseases, including known interactions with epigenetic changes, protein functional groups, DNA damage, increased DNA replication, and cell division [9–11]. Most of these associations have been demonstrated by mRNA expression assays [12, 13] and experimental data also have linked altered miRNAs expression with exposure Cd recently [11, 14].

Small RNAs may be involved in the regulation/signaling of metal toxicity response [14, 15]. miRNAs are a class of endogenous, noncoding, single-stranded, small RNA molecules of 19–22 nucleotides. miRNAs regulate expression of target genes at the posttranscriptional level by binding to 3′ untranslated regions of target mRNAs [16]. Moreover, the functional strand of mature miRNA loads onto the RNA-induced silencing complex (RISC) to suppress the expression of specific mRNA targets by directing mRNA degradation or translational repression [17, 18]. In addition, miRNAs also play a serious role in cell fate decisions; they are involved in the many vital biological events, including proliferation, differentiation, apoptosis, metabolism and viral infection, and many diseases including diseases of various organ systems, metabolic disorders, and cancer at several sites [19–21]. Many miRNAs have been proved to act as oncogenes and tumor suppressors [22].

miRNAs have widespread impact on expression and evolution of protein-coding genes [23]. To date, several computational methods have been successfully developed for predicting miRNAs and their targets [24, 25]. With regard to the species-specific miRNAs, they can be predicted depending on the intragenomic matching between miRNA candidates and their targets coupled with support vector machine classification of miRNA precursors. Inhibition efficiency of miRNAs to target genes mainly was determined by the 5′ end of a nucleotide sequence and site from 2 to 8.

The microarray analysis revealed that this metal modulated expression of nearly 400 genes in* Arabidopsis* and more than 1,700 in rice [26]. Recent research showed that cadmium stress affects the levels of miRNAs in soybean, rape plants, and rice [27–31]. Martínez-Pacheco et al. investigated the immortalized mouse fibroblast cell line BALB/3T3 A31-1-1 with exposed metal mixture including cadmium, which showed that the metal mixture resulted in miRNA expression profile that might be responsible for the mRNA expression changes observed under experimental conditions in which coding proteins were involved in cellular processes, including cell death, growth, and proliferation related to the metal-associated inflammatory response and cancer [32].

We previously established a model system of morphological cell transformation with cadmium chloride (CdCl_2_) in 16HBE [12]. This could provide in vitro human model system to examine the molecular events occurring of Cd carcinogenesis [12]. In the current study, Cd-transformed cells at the 35th passage were chosen as the treatment group and untreated normal 16HBE as the control group. In order to identify and study differentially expressed mRNAs and miRNAs after cadmium exposure, miRCURY LNA 7th miRNA chip and the Human mRNA Array v2.0 (8 × 60 K, Arraystar) were simultaneously used for high-throughput screening. And this can also provide theory evidence for further researching. After the related target genes were identified, bioinformatics methods of GO and pathway analysis were used.

## 2. Materials and Methods

### 2.1. Cell Culture and Treatments

16HBE cells were morphologically transformed using CdCl_2,_ as previously described [12]. Untransformed 16HBE cells (controls) and Cd-transformed cells at the 35th (5 *μ*mol L^−1^ Cd for 14 weeks) passage were cultured in RPMI-1640 containing L-glutamine (Sigma, St Louis, USA), 10% fetal bovine serum (FBS), and 1% penicillin/streptomycin (Life, California Carlsbad, USA) at 37°C in a 5% CO_2_ humidified atmosphere. The cells were passaged twice weekly and cells in logarithmic growth phase (2–5 × 10^5^ cells/mL) were harvested for following experiments.

### 2.2. RNA Isolation

Total RNA was harvested by using TRIzol (Invitrogen, California Carlsbad, USA) and miRNeasy mini kit (QIAGEN, Dusseldorf, Germany) according to manufacturer's instructions. The RNA quality was assessed on agarose gels and the concentration was determined with a NanoDrop spectrophotometer (ND-1000, Namedrops Technologies, Wilmington, DE, USA).

### 2.3. mRNA Expression Assays and Analysis

Microarray assays were performed by a service provider, Kangchen Bio-tech (http://www.kangchen.com.cn/). The sample preparation and microarray hybridization were performed based on the manufacturer's standard protocols with minor modifications. Briefly, mRNA was purified from total RNA after removal of rRNA (mRNA-ONLY Eukaryotic mRNA Isolation Kit, Epicentre, Wisconsin Madison, USA). Then, each sample was amplified and transcribed into fluorescent cRNA along the entire length of the transcripts without 3′ bias utilizing a random priming method. The labeled cRNAs were hybridized onto the Human mRNA Array v2.0 (8 × 60 K, Arraystar, Shanghai, China). For our experiments, scanned images were then imported into Agilent Feature Extraction software (version 11.0.1.1) for grid alignment and data extraction. Experiments were conducted in three 16HBE and three 35th 16HBE, and the 35th 16HBE were treated with different concentration of cadmium chloride (low toxicity, medium toxicity, and high toxicity).

Quantity normalization and subsequent data processing were performed by using the GeneSpring GX v11.5.1 software package (Agilent, California, USA). After quantile normalization of the raw data, mRNAs where at least 5 out of 6 samples had flags in present or marginal (“All Targets Value”) were chosen for further data analysis. Differentially expressed mRNAs were identified through fold change (fold change ≥ 2.0) and *t*-test (*p* values < 0.05) filtering.

### 2.4. miRNA Expression Assays and Analysis

MiRNA microarray assays were also performed by a service provider, Kangchen Bio-tech (http://www.kangchen.com.cn/). The 3,100 miRNA sequences were predicted by Lindow et al. The 7th generation of miRCURY LNA Array (v.18.0) (Exiqon, Copenhagen, Denmark) was used to identify Cd-responsive miRNAs on microarray chips. Microarray experiments were performed twice using distinct biological samples. Scanned images were then imported into GenePix Pro 6.0 software (Axon, Groningen, Holland) for grid alignment and data extraction. Replicated miRNAs were averaged and miRNAs with intensities ≥30 in all samples were chosen for calculating normalization factor. Expressed data were normalized using the Median normalization. After normalization, differentially detected signals were those with fold change ≥1.3 and *p* values < 0.05.

### 2.5. Bioinformatics Analysis of Data

Both mRNA and miRNA expression data were normalized with internal housekeeping sequences. The fluorescent signal intensity and background measurements were obtained on six spots for each mRNA. The results filtering and normalization were processed with the Microsoft Excel software, where the background value was subtracted from the fluorescence of the sample for both the control and experimental condition, and then compared between them to express the result regulation direction as fold change and *t*-test. Before comparing the differential expression of genes in response to Cd treatment, normalized gene expression levels were obtained by normalizing the number of raw clean tags in each library to the number of transcripts per million clean tags (TPM). A rigorous algorithm method was performed for the differential expression detection of genes across samples.

A combination of FDR < 0.001 (using Benjamini and Hochberg method; 1995) and the absolute value of log_2_Ratio ≥ 1 was used as the threshold to determine the significance of differentially expressed genes. Online databases like miRanda, TargetScan, and miRBase were chosen and searched to find predicted targets. In order to further identify differential miRNA genes, which may play potential roles in biological processes, the differentially expressed targets of specific miRNA were compared with the differentially expressed mRNA, and the intersection genes were defined as relevantly potential genes and involved in potential pathways. GO and pathway enrichment analysis were based on the gene ontology (http://www.geneontology.org/) and KEGG pathways (http://www.genome.jp/kegg/) [33–35].

### 2.6. Quantitation Real-Time RT-PCR of miRNAs and Target Gene

Total RNA (1 *μ*g) was isolated from untreated 16HBE cells, 35th Cd-induced 16HBE cells, and tumorigenic cells using TRIzol according to manufacturer's instructions. The purity and integrity of total RNA were analyzed by spectrophotometry and agarose gel electrophoresis, respectively. After purification, the miRNAs were reverse transcribed into cDNA with the specific RT primer by the Prime Script RT reagent Kit (Promega, China). qPCR was performed using PCR reagent Kit (Promega, China) according to manufacturer's instructions. The expression of CCM2 mRNA was detected with reverse transcription-polymerase chain reactions (RT-PCRs) using Reverse Transcription System (Promega: Madison, WI, USA). Gene expression levels were calculated based on the comparative quantitative method (the ΔΔCT method) [36]. The primers used in qRT-PCR were listed in Table 1.

## 3. Results

### 3.1. Identification of Cd-Responsive Novel Candidate mRNAs

**Figure 1:**
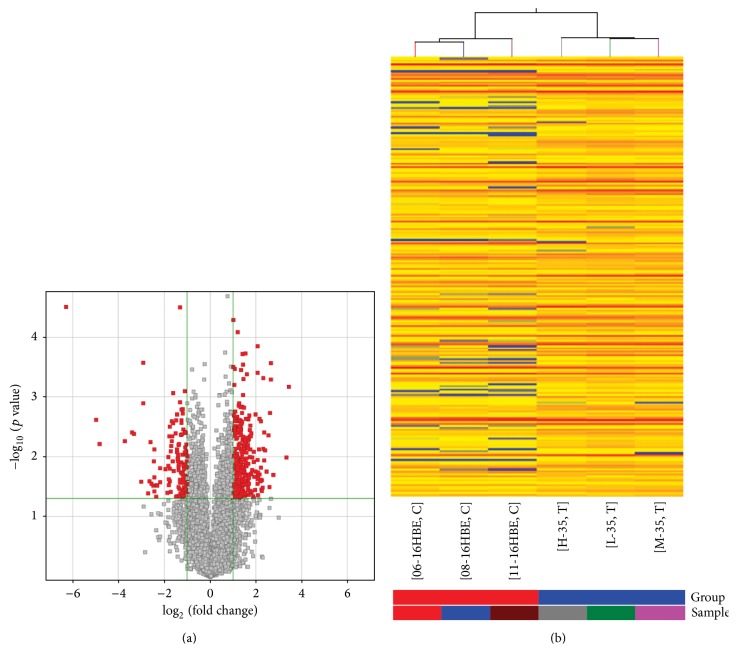
Differentially expressed mRNAs analysis. Volcano Plot (a) shows the relationship between fold change and statistical significance. The red points in the plot represent the differentially expressed mRNAs with statistical significance. The heat map diagram (b) shows cluster analysis of DEGs. The colors represents the expression values of DEGs. Sample names are listed in the horizontal axis, and high, medium, and low are 16HBE treated with different concentration of CdCl_2_ for 35 passaged. Right vertical axis represents the clustering of mRNAs.

With abundant and varied probes (30,215 mRNAs) in our microarray, of them, there were 366 mRNAs with upregulated expression and 132 with downregulated expression (≥2.0 fold change, *p* ≤ 0.05; see Supplementary Table S1 in the Supplementary Material available online at http://dx.doi.org/10.1155/2015/902025) in Cd-induced 35th cells when compared with untreated 16HBE cells. The Volcano Plot (Figure 1(a)) was applied to identify differentially expressed miRNAs with statistical significance. The heat map diagram (Figure 1(b), Supplementary Table S2) showed the result of the two-way hierarchical clustering of mRNAs and samples.

### 3.2. Identification of Cd-Responsive Novel Candidate miRNAs

**Figure 2:**
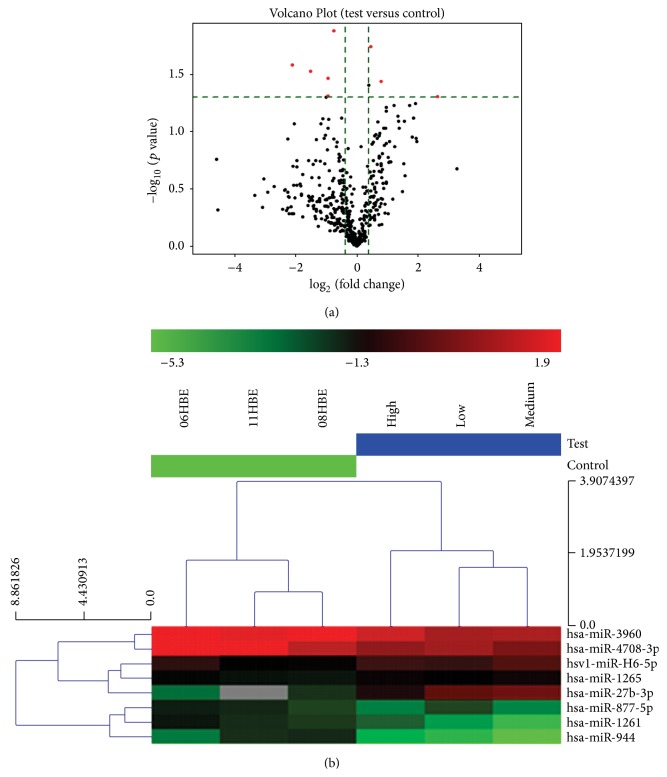
Differentially expressed miRNAs analysis. (a) Showing the relationship between fold change and statistical significance. The red points in the plot represent the differentially expressed miRNAs with statistical significance. (b) Showing cluster analysis of DEGs. The colors from green to red represent the fact that the expression values of DEGs become higher and higher. Sample names are listed in the horizontal axis, and high, medium, and low are 16HBE treated with different concentration of CdCl_2_ for 35 passaged. Right vertical axis represents the clustering of miRNAs.

To identify differentially expressed miRNAs with statistical significance, we performed Volcano Plot (Figure 2(a)) filtering between the two groups from the experiment. miRNAs with intensities ≥ 30 in all samples were chosen for calculating Median normalization factor. The threshold we used to screen up- or downregulated miRNAs is fold change ≥1.3 and *p* value ≤ 0.05. A total of 8 putative miRNAs (Supplementary Table S3) were detected to be Cd-responsive by microarray assays. They were considered to be new miRNAs and named according to the probe ID on the microarray chips as follows: hsa-miR-27b-3p, hsv1-miR-H6-5p, hsa-miR-1265, hsa-miR-944, hsa-miR-3960, hsa-miR-4708-3p, hsa-miR-877-5p, and hsa-miR-1261 (Table 2, Supplementary Table S5). hsa-miR-27b-3p, hsv1-miR-H6-5p, and hsa-miR-1265 were upregulated under Cd stress, whereas the other miRNAs were all downregulated. The heat map diagram (Figure 2(b), Supplementary Table S4) showed hsa-miR-27b-3p, hsv1-miR-H6-5p, and hsa-miR-1265 genes in Cd-induced 35th cells were higher (deeper) than in untreated 16HBE cells, suggesting that these differentially expressed genes in Cd-induced 35th cells were upregulated and this was consistent with the fact that 3 genes out of 8 differentially expressed genes were upregulated by differentially expressed genes analysis (*y*-axis and *x*-axis).

### 3.3. Analysis of MicroRNAs to Target mRNAs

**Figure 3:**
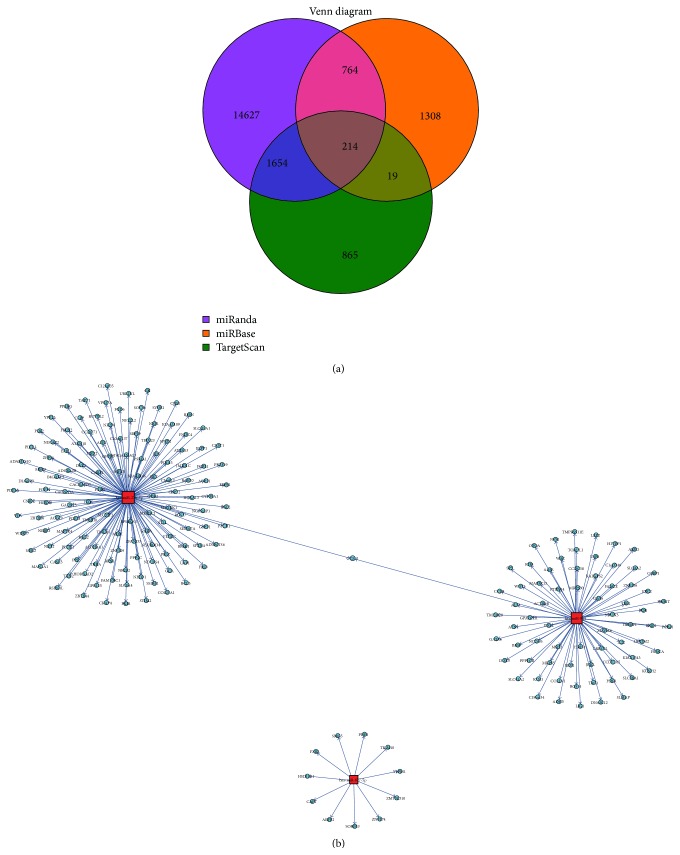
Targets of DEGs. (a) Venn diagrams showing the unique and shared regulated targets in DEGs; (b) showing the networks of 214 microRNA-mRNA interaction pairs of three of the databases.

In our further analysis, we focused on the trend of expression changes of miRNA and its target genes. In order to identify correlations between them, we searched three online databases to find predicted targets in the miRanda (http://www.microrna.org/), TargetScan (http://www.targetscan.org/), and miRBase (http://www.mirbase.org/). There were 17,259 miRNA-mRNA pairs in miRanda, 2,305 pairs in miRBase, and 2,752 pairs in TargetScan (Supplementary Table S5). A Venn diagram (Figure 3(a), Supplementary Table S5) showed that 214 of the microRNA-mRNA interaction pairs were acquired from three of the databases. The network figure (Figure 3(b)) showed the network relationship between miRNA and mRNA. CCM2 is the common target between hsa-miR-27b-3p and hsa-miR-944.

### 3.4. Combination Analysis of MicroRNA Microarray and mRNA Microarray

According to the combination analysis, three miRNAs were found in the intersection, including hsa-miR-27b-3p, hsa-miR-944, and has-miR-877-5p. Only the two former miRNAs could regulate same genes in mRNA microarray, especially has-miR-27b-3p. The details of genes were shown in Table 3.

### 3.5. GO Analysis of DEGs

**Figure 4:**
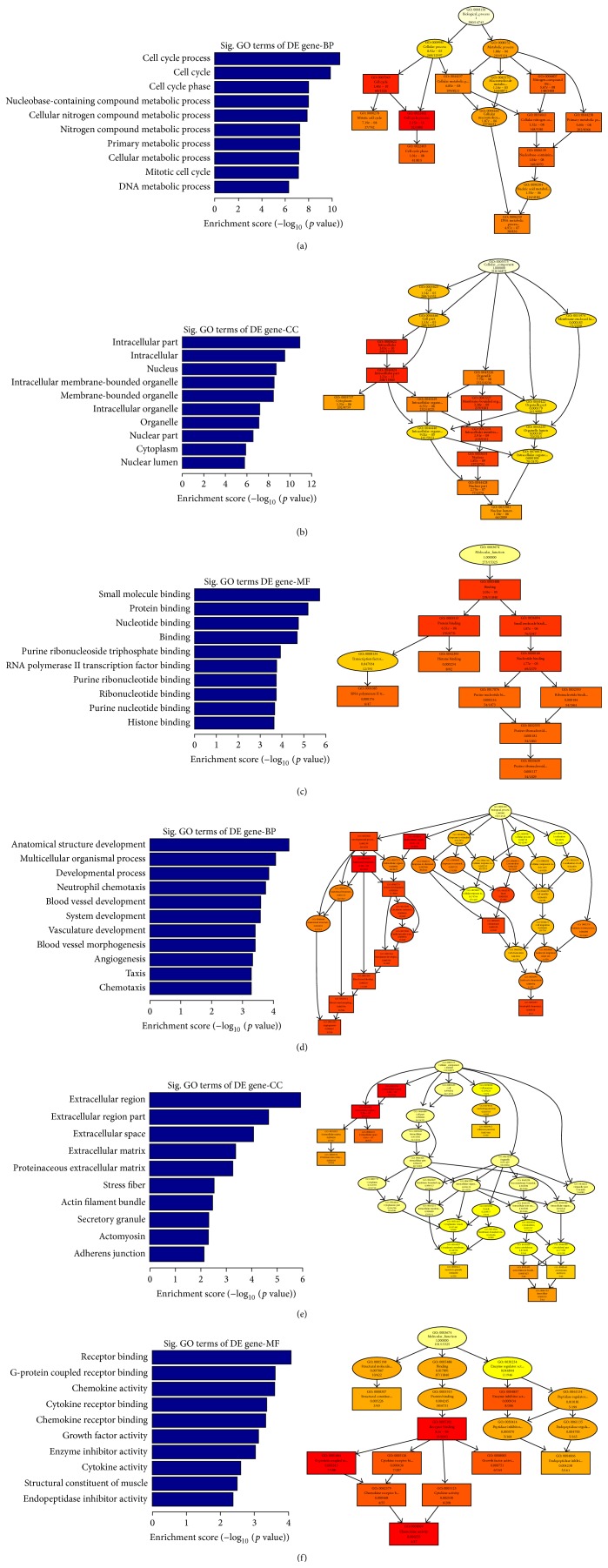
GO analysis of differentially expressed mRNAs in response to Cd stress. The top 10 of the most related parts were shown (a–f); (a–c) showed biological process (a), cellular component (b), and molecular function (c) of upregulated mRNAs, respectively. (d–f) showed biological process (d), cellular component (e), and molecular function (f) of downregulated mRNAs.

**Figure 5:**
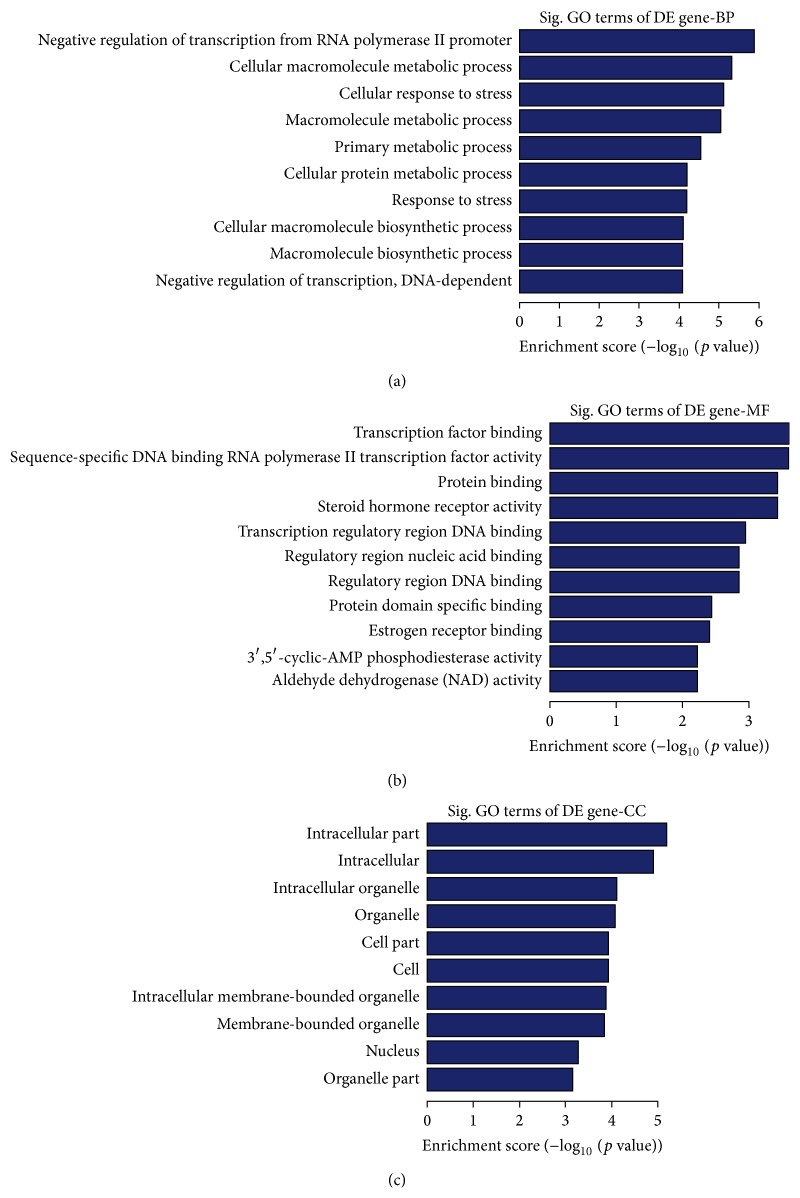
GO analysis of differentially expressed miRNA targets in response to Cd stress. The top 10 of the most related parts were shown (a–c); biological process (a), cellular component (b), and molecular function (c).

For GO analysis, the ten most related genes and pathways were used. As shown by Figure 4 (Supplementary Table S6), GO analysis showed that the aberrant mRNA expression genes took part in many biological processes: primary metabolism, cell metabolism, cell cycle progression, DNA damage and repair, biological cycle, and so forth. GO analysis showed that the targets of aberrant miRNA took part in many biological processes, such as primary metabolism, cellular response to stress, negative regulation of transcription, DNA-dependent, and regulation of endocrine process (Figure 5, Supplementary Table S7). These results supported the idea that Cd-induced malignant transformation was related to DNA damage and repair and biological cycle.

### 3.6. Pathway Analysis of DEGs

**Figure 6:**
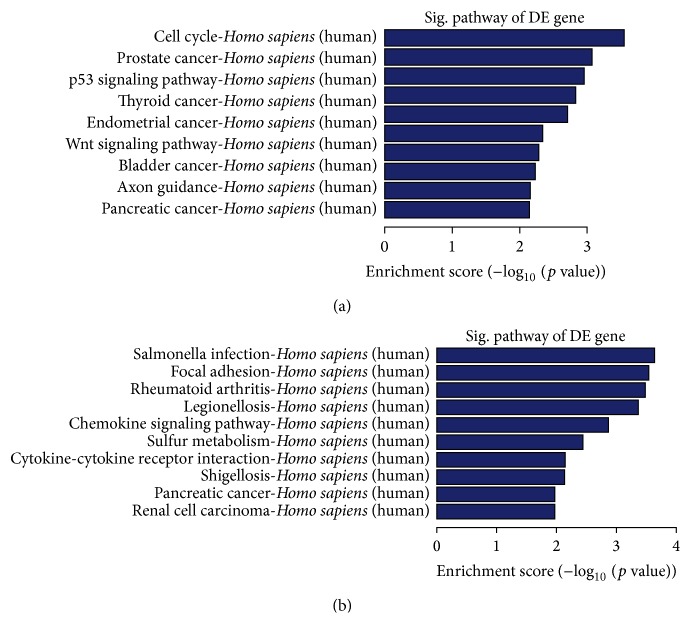
The KEGG pathway analysis of differentially expressed mRNAs. Pathways with *p* value less than 0.05 were shown in the figure. All the *p* values were transformed into log *p* value.

For the KEGG pathway analysis, pathways with *p* value less than 0.05 were used. Pathway analysis of differentially expressed mRNAs (Figure 6, Supplementary Table S8) showed that they were involved in the cell cycle, P53 signaling pathway, prostate cancer, thyroid cancer, and endometrial cancer, Wnt signaling pathway, glioma, bladder cancer, pancreatic cancer, and axon guidance.

### 3.7. Expression Analysis of has-miR-27b-3p, has-miR-944, and Their Common Target Gene in Cd-Induced 16HBE Cells

**Figure 7:**
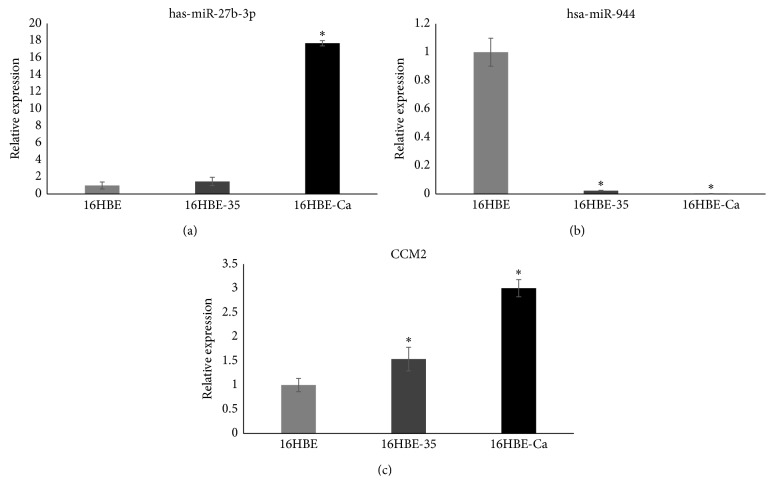
The expression of has-miR-27b-3p, has-miR-944, and CCM2 by qRT-PCR in different groups. *∗* represents *p* < 0.05 as compared to 16HBE, respectively.

A significant upregulated expression of has-miR-27b-3p and CCM2 in malignant 16HBE cells (16HBE cells, 35th Cd-induced 16HBE cells, and tumorigenic cells) was found, while has-miR-944 was downregulated in malignant 16HBE cells. The trend was aggravated as the cells passage (Figure 7). The results are consistent with microarrays.

## 4. Discussion

At present, lots of studies have confirmed molecular mechanisms of cadmium carcinogenesis including inhibit apoptosis, DNA repair and DNA methylation, histone acetylation [9, 11, 37], reactive oxygen species in cadmium response, and cadmium-induced signal transduction pathways [38]. In recent years, microRNAs have become the research focus in the scientific field. It is identified that about 50% of human microRNA genes are located at fragile sites and genomic regions involved in cancers [39], which clarifies the important role of miRNA in the tumorigenesis. Global expression profiling analysis of miRNAs and mRNAs in the same samples may provide a unique opportunity to enhance our understanding of potential miRNA regulatory mechanisms in 16HBE cells which were exposed to Cd. In this study, these altered expression profiles of mRNA and miRNA were screened by mRNA and miRNA microarray after 16HBE cell exposed cadmium. To further validate the regulatory role of miRNAs in Cd-responsive cells, the corresponding target mRNAs of Cd-regulated miRNAs were predicted by intersection of the database of miRanda, TargetScan and miRBase. 214 target mRNAs of 3 miRNAs were identified by bioinformatics prediction (Table 1, Supplementary Table S5). 133 genes were found as the target genes of hsa-miR-27b-3p and 11 genes were found as the target genes of hsa-miR-877-5p; however 70 genes were found as the target genes of hsa-miR-944. CCM2 was the same target gene of hsa-miR-27b-3p and hsa-miR-944. We also found that other miRNAs were related with CCM2 in these databases, miRanda, TargetScan, and miRBase. However the target genes of two miRNAs in TargetScan are CCM2, while more than two miRNAs' target genes are CCM2 in the nother two databases, both of them including hsa-miR-27b-3p, hsa-miR-877-5p, and hsa-miR-944 (Supplementary Table S5). The combination analysis could narrow down the range of miRNA candidates and clearly explicit the biological functions of target genes.

Most of miRNAs are clustered and shared similar expression patterns, implying that they are transcribed as polycistronic transcripts [40, 41]. Further research found the mutations of CCM2 gene lead to cerebral cavernous malformations. CCM2 encodes a scaffold protein that functions in the stress-activated p38 mitogen-activated protein kinase (MAPK) signaling cascade. The protein interacts with SMAD (small mothers against decapentaplegic) specific E3 ubiquitin protein ligase 1 (also known as SMURF1) via a phosphotyrosine binding domain to promote RhoA degradation. The protein is required for normal cytoskeletal structure, cell-cell interactions, and lumen formation in endothelial cells. Multiple transcript variants encoding different isoforms have been found for this gene (provided by RefSeq, Nov 2009). The analysis of GO showed differently expressed miRNAs of Cd-responsive cells were reported to be involved in several biological processes, including negative regulation of transcription from RNA polymerase II promoter, cellular macromolecule metabolic process, and cellular response to stress, involved in several molecular functions transcription factor binding, ligand-activated sequence-specific DNA binding RNA polymerase II transcription factor activity, and protein binding. Those illustrated miRNAs are transcribed by RNA polymerase II as primary miRNAs (pri-miRNAs), which is consistent with the research of Cai et al., 2004 [42], Lee et al., 2004 [43], and so forth.

It is shown that cadmium can upregulate genes of encoding pathogen related proteins, antioxidant enzymes, transporters, TFs, and proteins associated with glutathione metabolism. With the literature supporting and confirmation, we show the importance of miRNAs in key cellular processes by modulating a small gene set in this study. Together, our findings allow us to assert the great importance of miRNAs in the development and/or establishment of cadmium-associated diseases, especially cancer, through key cellular processes modulated by cadmium exposure targets that have also been cited as above. The patterns of mRNA and miRNA expression that predict establishment of cadmium health effects are helpful for understanding the aberrant molecular mechanisms of human cells. However, further study is needed to understand the relationship between genetic predisposition and epigenetic disruption in the development of cadmium-associated diseases.

In summary, our results provide a tight link for the miRNA-mRNA integrated network after 16HBE exposed Cd. These results provide some new and meaningful candidate miRNAs and genes to allow further investigation of the mechanism after 16HBE differentiation and to provide several important molecular markers for the diagnosis and therapy of related diseases. Further study of the potential function of miRNA-mRNA integration in Cd-response will promote our understanding of the key role of 16HBE in Cd-response, such as further research of differentially expressed genes demonstrated by Western Blot, or various methods about the target gene CCM2 and genes of combination analysis.

## Supplementary Material

S1 lists the DEGs of mRNA microarray in Cd-induced 35th cells when compared with untreated 16HBE cells, which 361 mRNAs were upregulated and 127 were downregulated.

## Figures and Tables

**Table 1:** Sequences of primers used in qRT-PCR detection.

Primer	Sequence	Length
has-miR-27b-3p		
Forward	5′-ACACTCCAGCTGGG TTCACAGTGGCTAAG-3′	98
Reverse	5′-ATCCAGTGCAGGGTCCGAGG-3′	
RT primer	CTCAACTGGTGTCGTGGAGTCGGCAATTCAGTTGAG GCAGAACT	

has-miR-944		
Forward	5′-GCGGCGGAAATTATTGTACATC-3′	98
Reverse	5′-ATCCAGTGCAGGGTCCGAGG-3′	
RT primer	GTCGTATCCAGTGCAGGGTCCGAGGTATTCGCACTGGATACGACCTCATC	

CCM2		
Forward	5′-GAGACCATTGGCGTGAAGGA-3′	177
Reverse	5′-GATGTCCGAGATCATGCGGT-3′	

**Table 2:** Predicted targets of Cd-responsive miRNAs in human bronchial epithelial cells

miRNAs	Target genes
*Upregulated *	
hsa-miR-27b-3p	CCM2, ADAMTS10, B4GALT3, C12orf35, DLGAP3, EML1, FAM108C1, GALNT5, HORMAD2, KIAA0146…
hsa-miR-1265	ABAT, ELAVL2, IKZF4, MAGIX, PPAP2B, XPNPEP2…
*Downregulated *	
hsa-miR-877-5p	CCM2, ARFIP2, CAP1, FXR2, HSD11B1, PHF8, SMG5, SORBS3, TRIM10…
hsa-miR-944	CCM2, AASS, BRIP1, C18orf34, DDX5, EPC2, FANCE, GAS6, HIVEP1, KCNH2, ZMYM6…
hsa-miR-1261	AQP4, BRCA1, CCDC85C, EIF2A, EIF4E3, EIF4G2, FRMPD4, SUPT7L…
hsa-miR-3960	C14orf43, HOXB8, MARVELD1, PCDHA8, PCDHA12, SLC9A3…
hsa-miR-4708-3p	BAZ2B, ATP2A3, C12orf53, EFNB1, EIF2S2, EIF4EBP2…

**Table 3:** Combinational analysis in the data of microRNA and mRNA microarray.

	hsa-miR-27b-3p	hsa-miR-944
Up/down	Up	Down
mRNAs		
Upregulated	RGS17, ITSN2, PRPF19, and PLK2	PPP1CB
Downregulated	SYDE1	DNAJC12

## Abbreviations

16HBE: Human bronchial epithelial cells
Cd: Cadmium
CdCl_2_: Cadmium chloride
CO_2_: Carbon dioxide
CIP: Calf Intestine Phosphatase
CCM2: Cerebral cavernous malformations 2
DEGs: Differently expression genes
FDR: The false discovery rate
GO: Gene ontology
IARC: The International Agency for Research on Cancer
KEGG: Kyoto Encyclopedia of Genes and Genomes
miRNA: MicroRNA
mRNA: Messenger RNA
MAPK: Mitogen-activated protein kinase
RISC: RNA-induced silencing complex
SMAD: Small mothers against decapentaplegic
TPM: Transcripts per million clean tags.
