# Supplementary material for: MicroRNAs-mRNAs Expression Profile and Their Potential Role in Malignant Transformation of Human Bronchial Epithelial Cells Induced by Cadmium
Source: Biomed Res Int. 2015 Oct 4;2015:902025. doi: 10.1155/2015/902025 (PMC4609416; doi:10.1155/2015/902025)
Supplement: Supplementary file 1 — S1 lists the DEGs of mRNA microarray in Cd-induced 35th cells when compared with untreated 16HBE cells, which 361 mRNAs were upregulated and 127 were downregulated. [file 902025.f1.zip › 902025.r3.attachment/supplementary materials/RNA_QC.pdf]

# RNA QC EUK

## 1. RNA Quantification and Quality Assurance by NanoDrop ND-1000

| Sample ID | OD260/280 Ratio | OD260/230 Ratio | Conc. (ng/µl) | Volume (µl) | Quantity (ng) | QC result Pass or Fail |
|---|---|---|---|---|---|---|
| L-35 | 2.02 | 2.42 | 1402.94 | 30 | 42088.20 | pass |
| M-35 | 2.03 | 2.46 | 1090.73 | 30 | 32721.90 | pass |
| H-35 | 2.00 | 2.45 | 1100.83 | 50 | 55041.50 | pass |
| 06-16HBE | 2.01 | 2.42 | 814.06 | 80 | 65124.80 | pass |
| 08-16HBE | 2.00 | 2.42 | 752.08 | 80 | 60166.40 | pass |
| 11-16HBE | 1.99 | 2.46 | 717.18 | 100 | 71718.00 | pass |

*For spectrophotometer, the O.D. A260 /A280 ratio should be close to 2.0 for pure RNA (ratios between 1.8 and 2.1 are acceptable). The O.D. A260/A230 ratio should be more than 1.8.

## 2. RNA Integrity and gDNA contamination test by Denaturing Agarose Gel Electrophoresis

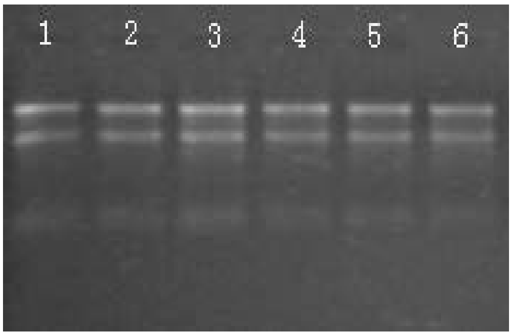


| | | |
|---|---|---|
| Lane 1: | Total RNA of sample | L-35 |
| Lane 2: | Total RNA of sample | M-35 |
| Lane 3: | Total RNA of sample | H-35 |
| Lane 4: | Total RNA of sample | 06-16HBE |
| Lane 5: | Total RNA of sample | 08-16HBE |
| Lane 6: | Total RNA of sample | 11-16HBE |

*The 28S and 18S ribosomal RNA bands should be fairly sharp, intense bands. The intensity of the upper band should be about twice that of the lower band. Smaller, more diffuse bands representing low molecular weight RNAs (tRNA and 5S ribosomal RNA) may be present. It is normal to see a diffuse smear of ethidium bromide staining material migrating between the 18S and 28S ribosomal bands, probably comprised of mRNA and other heterogeneous RNA species. DNA contamination of the RNA preparation will be evident as a high molecular weight smear or band migrating above the 28S ribosomal RNA band. Degradation of the RNA will be reflected by smearing of ribosomal RNA bands.

KangChen Bio-tech 421 Hong Cao Rd. Building 63, 2nd floor Shanghai P.R. China 200233
Tel: 86-21-64451989 Fax: 86-21-64452021
Email: ping@kangchen.com.cn Website: www.kangchen.com.cn

## 3. Labeling Efficiency-QC

In the experiment, 1μg RNA is used for labeling. The specific activity (pmol dyes per μg cRNA) of the labeled RNA can be obtained by the following calculation:

$$\text{Specific Activity} = \frac{(\text{pmol per } \mu\text{l dye})}{(\mu\text{g per } \mu\text{l cRNA})}$$

| Sample ID | Dye Name | Dye pmol/μl | cRNA Concentration (μg/μl) | Specific Activity*(pmol Dye/μg cRNA) | Volume (μl) | Total Amount (μg) |
|---|---|---|---|---|---|---|
| L-35 | Cy3 | 16.78 | 0.83644 | 20.06121 | 20 | 16.7288 |
| M-35 | Cy3 | 16.46 | 0.78433 | 20.98606 | 21 | 15.6866 |
| H-35 | Cy3 | 16.75 | 0.78447 | 21.352 | 22 | 15.6894 |
| 06-16HBE | Cy3 | 17.21 | 0.79569 | 21.62903 | 23 | 15.9138 |
| 08-16HBE | Cy3 | 16.3 | 0.74853 | 21.77601 | 24 | 14.9706 |
| 11-16HBE | Cy3 | 16.27 | 0.79659 | 20.42456 | 25 | 15.9318 |

*For two-color, if the yield is <825 ng and the specific activity is <8.0 pmol Cy3 or Cy5 per μg cRNA do not proceed to the hybridization step. Repeat cRNA preparation.

*For one-color, if the yield is <1.65μg and the specific activity is <9.0 pmol Cy3 or Cy5 per μg cRNA do not proceed to the hybridization step. Repeat cRNA preparation.
