# Supplementary figures and images for: MicroRNAs-mRNAs Expression Profile and Their Potential Role in Malignant Transformation of Human Bronchial Epithelial Cells Induced by Cadmium
Source: Biomed Res Int. 2015 Oct 4;2015:902025. doi: 10.1155/2015/902025 (PMC4609416; doi:10.1155/2015/902025)

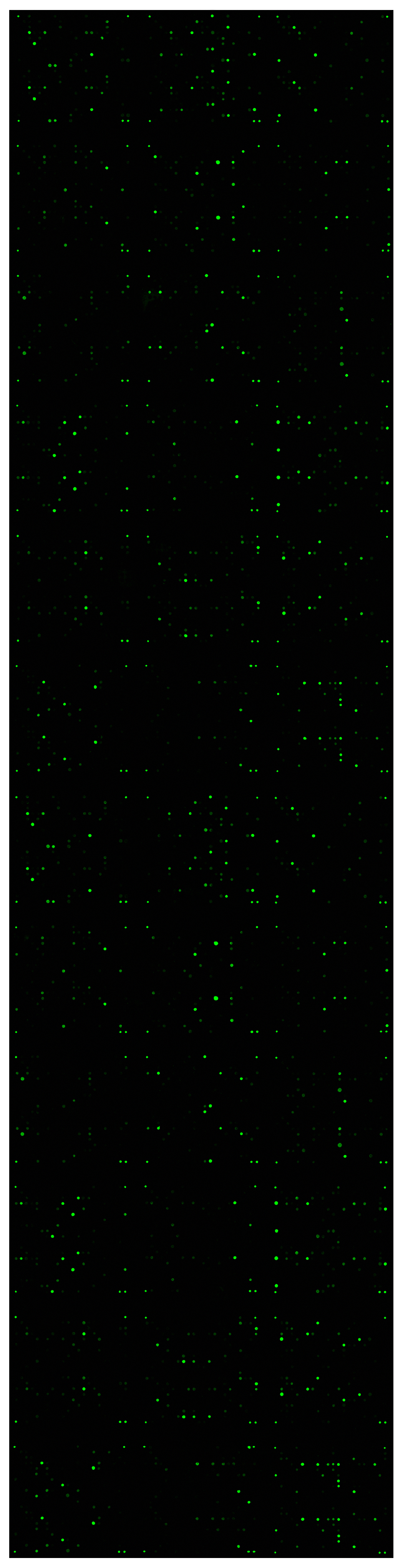

Supplement: Supplementary file 1 — S1 lists the DEGs of mRNA microarray in Cd-induced 35th cells when compared with untreated 16HBE cells, which 361 mRNAs were upregulated and 127 were downregulated. [file 902025.f1.zip › 902025.r3.attachment/supplementary materials/File 3. Graphs/06HBE.jpg]

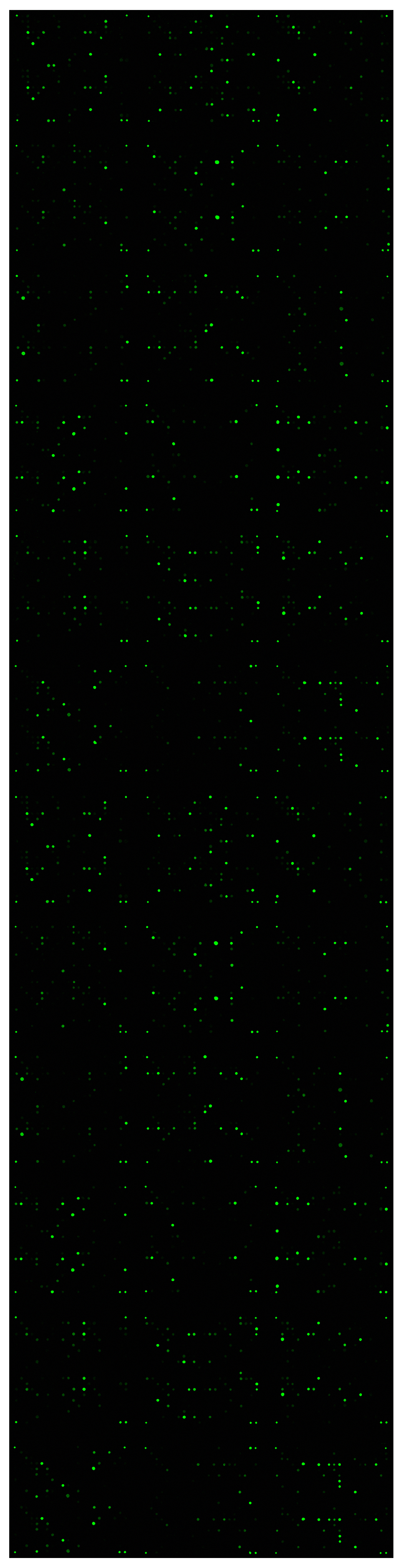

Supplement: Supplementary file 1 — S1 lists the DEGs of mRNA microarray in Cd-induced 35th cells when compared with untreated 16HBE cells, which 361 mRNAs were upregulated and 127 were downregulated. [file 902025.f1.zip › 902025.r3.attachment/supplementary materials/File 3. Graphs/08HBE.jpg]

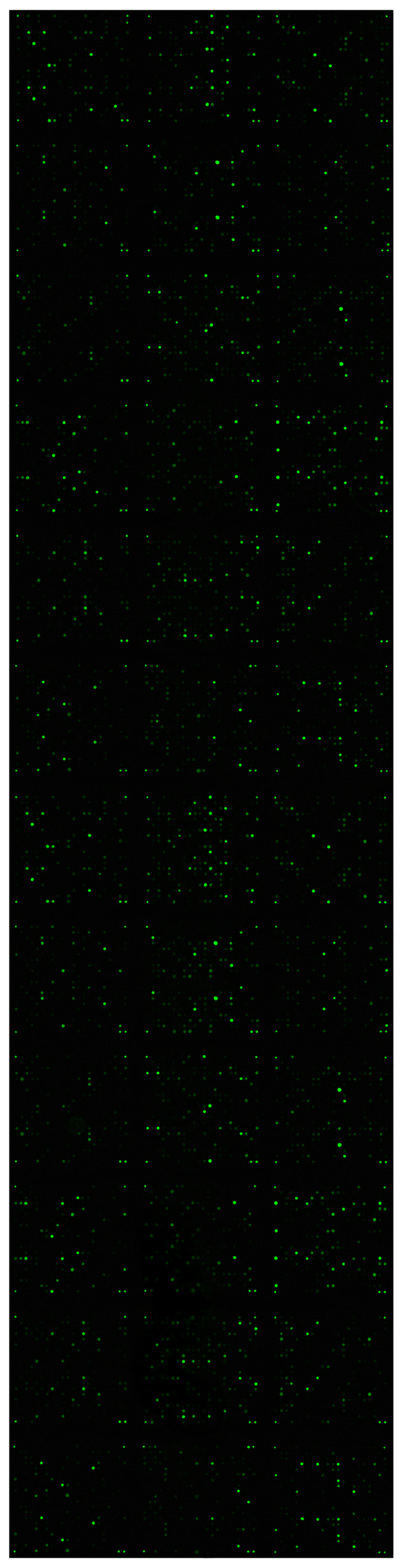

Supplement: Supplementary file 1 — S1 lists the DEGs of mRNA microarray in Cd-induced 35th cells when compared with untreated 16HBE cells, which 361 mRNAs were upregulated and 127 were downregulated. [file 902025.f1.zip › 902025.r3.attachment/supplementary materials/File 3. Graphs/11HBE.jpg]

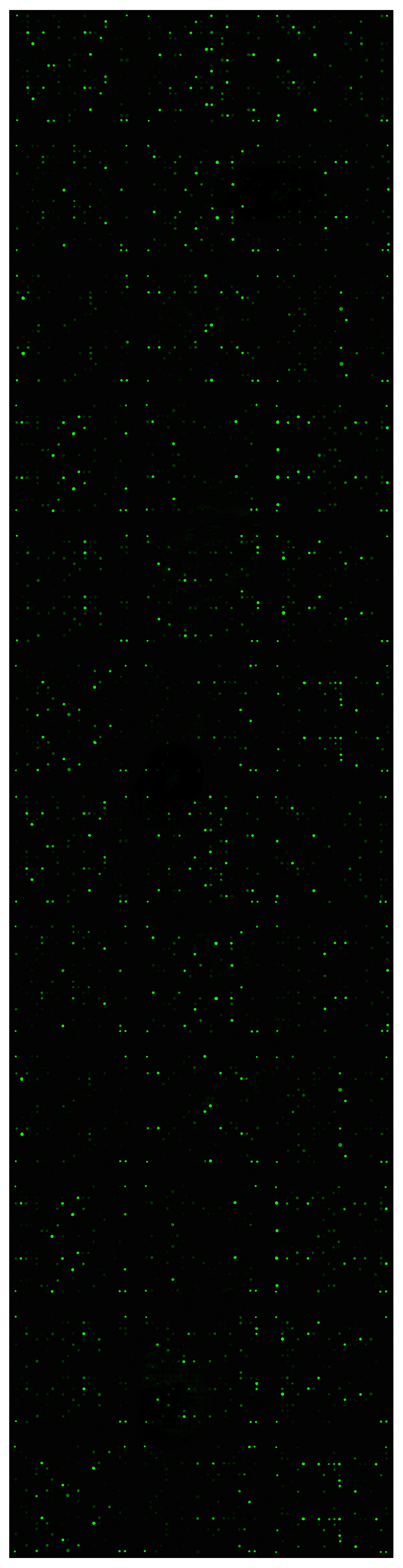

Supplement: Supplementary file 1 — S1 lists the DEGs of mRNA microarray in Cd-induced 35th cells when compared with untreated 16HBE cells, which 361 mRNAs were upregulated and 127 were downregulated. [file 902025.f1.zip › 902025.r3.attachment/supplementary materials/File 3. Graphs/high.jpg]

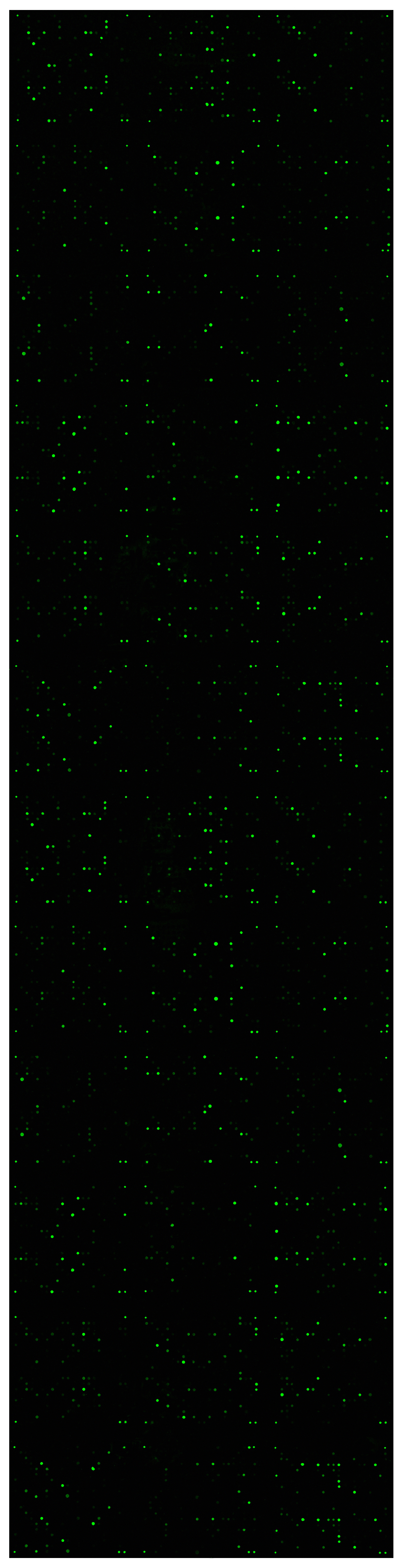

Supplement: Supplementary file 1 — S1 lists the DEGs of mRNA microarray in Cd-induced 35th cells when compared with untreated 16HBE cells, which 361 mRNAs were upregulated and 127 were downregulated. [file 902025.f1.zip › 902025.r3.attachment/supplementary materials/File 3. Graphs/low.jpg]

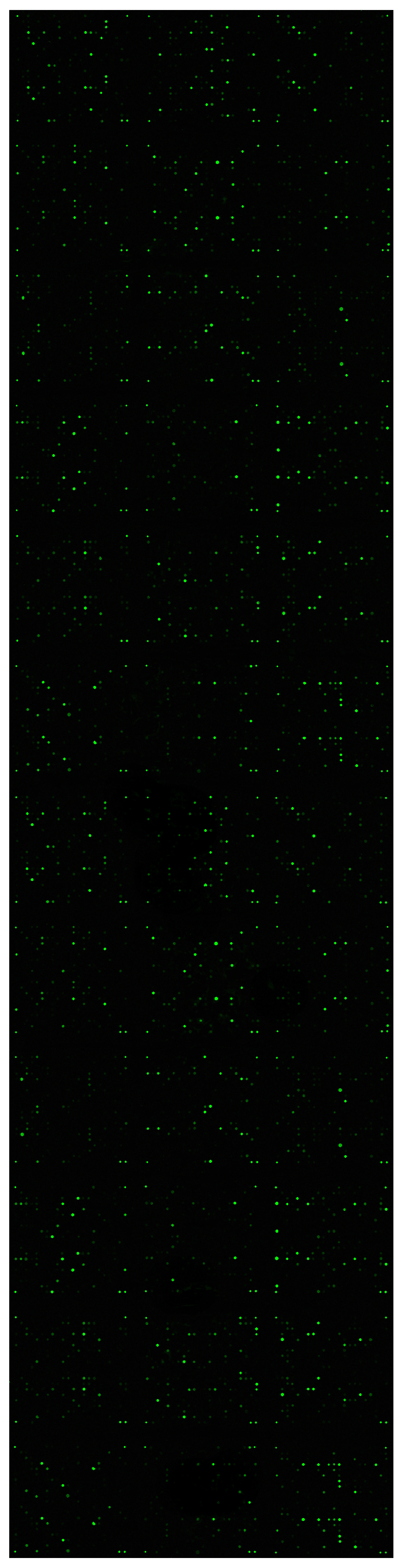

Supplement: Supplementary file 1 — S1 lists the DEGs of mRNA microarray in Cd-induced 35th cells when compared with untreated 16HBE cells, which 361 mRNAs were upregulated and 127 were downregulated. [file 902025.f1.zip › 902025.r3.attachment/supplementary materials/File 3. Graphs/medium.jpg]
